# Low-Temperature Stress-Induced Changes in Cucumber Plants—A Near-Infrared Spectroscopy and Aquaphotomics Approach for Investigation

**DOI:** 10.3390/s25247602

**Published:** 2025-12-15

**Authors:** Daniela Moyankova, Petya Stoykova, Petya Veleva, Nikolai K. Christov, Antoniya Petrova, Krasimir Rusanov, Stefka Atanassova

**Affiliations:** 1 AgroBioInstitute, Agricultural Academy, 1164 Sofia, Bulgaria; nikolai_christov@abi.bg (N.K.C.); krusanov@abv.bg (K.R.); 2 Department of Agricultural Engineering, Faculty of Agriculture, Trakia University, 6000 Stara Zagora, Bulgaria; petya.veleva@trakia-uni.bg (P.V.); antoniya.petrova@trakia-uni.bg (A.P.); 3 Centre of Competence “Sustainable Utilization of Bio-resources and Waste of Medicinal and Aromatic Plants for Innovative Bioactive Products” (BIORESOURCES BG), 1000 Sofia, Bulgaria

**Keywords:** near-infrared spectroscopy, aquagrams, cucumber plants, cold stress

## Abstract

Low temperatures have a significant impact on the growth, development, and productivity of cucumber plants. The potential of near-infrared spectroscopy and the aquaphotomics approach for investigating chilling stress was studied in Voreas F1 and Gergana cultivars. Changes in the spectral patterns of cucumber plants were compared with physiological and metabolic data. Voreas plants were unable to survive seven days of low-temperature stress due to a drastic increase in electrolyte leakage and a decrease in the net photosynthesis rate, stomatal conductance, and transpiration rate. Gergana plants survived chilling by preserving cell membrane integrity and photosynthesis efficiency. During chilling treatment, the content of most metabolites in both cultivars was reduced compared to the controls, yet it was much more pronounced in Voreas. We observed an increased accumulation of cinnamic acid on the seventh day only in the Gergana cultivar. A MicroNIR spectrometer was used for in vivo spectral measurements of cotyledons and the first two leaves. Differences in absorption spectra were observed among control, stressed, and recovered plants, across different days of stress, and between the studied cultivars. The most significant differences were in the 1300–1600 nm range, much smaller for Gergana than Voreas. Aquagrams of the two cultivars also reveal differences in their responses to low temperatures and changes in water molecular structure in the leaves. The errors of prediction for the days of chilling by using PLS models were from 0.96 to 1.14 days for independent validation, depending on the spectral data of different leaves used. Near-infrared spectroscopy and aquaphotomics can be used as additional tools for early detection of stress and investigation of low-temperature tolerance in cucumber cultivars.

## 1. Introduction

Plants’ productivity can be significantly affected by abiotic stresses, such as temperature extremes, high salinity, drought, nutrient depletion, or soil contamination. These factors influence plant growth, yield, and physiological functions. Cucurbitaceae family crops have been increasingly affected by unfavorable environmental conditions, and among them, cucumber (*Cucumis sativus* L.) appears to be the most sensitive cucurbit [1].

Cucumber is an economically important vegetable suitable for fresh and processed food. In 2023, it was cultivated worldwide on approximately 2.2 million hectares, and annual global cucumber production was approximately 97.8 million tons [2]. Cucumbers originated from tropical regions [3] and are cold-sensitive plants with an optimal growth temperature of 15–25 °C [4]. Low temperatures in early spring and autumn are a limiting environmental factor for cucumber germination, plant growth, yield, and areas of cultivation [4], which is exacerbated by climate change. Cold stress can change the structure of the cell membrane, reduce enzyme activity and metabolic capacity, etc., which have effects on the normal growth and development of plants [5,6,7,8,9]. Under cold stress, plant cells produce various reactive oxygen species, such as hydrogen peroxide, hydroxide ions, hydroxyl radicals, and superoxide anions, which can damage the cells [10,11]. Jung et al. [12] reported chilling injury on the photosynthesis system of strawberries. The stress caused by low temperatures and high humidity in greenhouses was investigated by Amin et al. [13]. The authors reported that stress factors affected the photosynthetic efficiency of cucumber plants, their antioxidant defense system, reactive oxygen species, and hormone profile. Plants in different developmental stages respond differently to stressful conditions, with phase 6 leaves showing greater tolerance than those in phase 2 and 4 leaves.

Optical sensors in the visible and near-infrared (NIR) regions are increasingly used for plant stress detection. Healthy plant leaves exhibit low reflectance in the visible region due to strong absorption by chlorophyll and other pigments, while in the near-infrared region, they have high reflectance due to light scattering caused by internal leaf structures. In the 1300–2500 nm region, there are two strong water absorption peaks at 1450 and 1940 nm. Absorption in the NIR region provides information about the chemical composition of plants [14,15]. Changes in the chemical composition and structure of cells, caused by different stress factors, would change the spectral characteristics of plant tissues in a specific way. Leaf anatomical properties, such as thickness and surface texture, also change under stress. Using different chemometric methods, it is possible to extract information from plants’ NIR spectra, create equations to determine quantitative parameters, and build classification models. NIR spectroscopy has multiple advantages over conventional chemical methods for plant analysis. It requires little or no sample preparation and can also be used for in-field measurements. Various chemical components or properties of plants can be determined with a single spectral measurement. This way, a particular combination of stress-induced chemical and physiological alterations in the plant organism can be correlated with a specific spectral pattern measured. Reviews of recently developed optical technologies, sensors, and their applications for diagnosing abiotic and biotic stress in plants have been published [16,17,18,19,20]. Several authors reported investigations connected to cold stress. The hyperspectral imaging system in the range of 410–990 nm was used to predict cold stress levels in strawberry plants [12]. The study provided scientific insights into the application of canopy remote sensing diagnostics of strawberry photosynthetic physiological chilling injury in practical agricultural production. The obtained accuracy of stress level prediction was 71.25% in validation samples. Choi et al. [21] reported estimation of the chilling injury severity of kimchi cabbage (*Brassica rapa* L. ssp. pekinensis), treated at several temperatures (0 °C, −1 °C, and −3 °C), based on hyperspectral images. Classification based on ANN achieved overall accuracies above 0.924 for four classes (normal, slight, moderate, and severe chilling injury). Pipatsart et al. [22] reported the successful application of hyperspectral images in the range of 874–1734 nm for the detection of chilling injuries in fresh coriander. Aquaphotomics is a novel scientific approach that utilizes near-infrared spectroscopy to investigate the molecular structure of water and its interactions within biological systems [23,24,25]. In agriculture, it offers several innovative applications. Aquaphotomics enables real-time monitoring of plant water status, stress responses, and nutrient uptake by analyzing water spectral patterns. A combination of NIRS with aquaphotomics has already been used for nondestructive investigation of plant abiotic stress and diseases. This new approach provides additional information about the plant’s growth conditions and health. NIR spectroscopy and the aquaphotomics approach for rapid in vivo diagnosis of virus-infected soybeans were reported by Jinendra et al. [26]. The study emphasizes the significance of water-related spectral responses in understanding plant disease dynamics and highlights the potential of NIR spectroscopy and aquaphotomics in agricultural diagnostics. Early detection of cold stress response in soybean cultivars with different stress tolerances was reported by Muncan et al. [27]. The investigation used near-infrared spectroscopy to analyze soybean leaves from five cultivars under optimal (27 °C) and cold stress (22 °C) conditions. The authors reported significant differences in NIR spectral profiles between plants in normal and cold stress conditions. The classification accuracy for detecting cold stress using the Soft Independent Modeling of Class Analogy (SIMCA) method was 100%, indicating strong separation between stressed and non-stressed plants. Aquagrams illustrated the differences in spectral patterns of soybean leaves under stress. The findings underscored the effectiveness of NIR spectroscopy and aquaphotomics in detecting cold stress in soybean plants, as well as contributing to our understanding of cold tolerance mechanisms, and offer a basis for developing rapid, non-destructive field methods for stress detection. An aquaphotomics approach was used to investigate changes in maize plants during water stress [28]. Differences in absorption spectra in the first overtone water region from 1300 to 1600 nm were observed between control, water-stressed, and recovered maize plants, as well as between different days of stressed plants. Aquagrams clearly visualized the spectral differences and showed changes in water molecular structure during water stress. Several authors applied aquaphotomics for monitoring of lettuce freshness [29] and strawberry fruit [30] during cold storage.

The main objective of this study was to evaluate the potential of near-infrared spectroscopy and the aquaphotomics approach for investigating low-temperature stress in cucumber plants by comparing results from changes in physiological parameters and metabolic content with those observed in spectral patterns.

## 2. Materials and Methods

### 2.1. Plant Materials and Experimental Conditions

Two cucumber (*Cucumis sativus* L.) cultivars, Gergana (Albena, Bulgaria) and Voreas F1 (Enza Zaden, Enkhuizen, The Netherlands), known for their fast growth, high yield, and fruits with excellent taste, were included in the study. Seeds were surface-sterilized with 5% commercial bleach (Domestos) for 10 min, washed three times with distilled water, and then placed on wet filter paper. After germination, the seedlings were transferred to a pot (d 10 cm) with soil. Plants were grown well-watered at 28/25 °C day/night temperature, 50 ± 5% relative air humidity, and at 150 µmol m^−2^ s^−1^—photon flux density of light intensity for 16 h in a growth chamber (MLR-351, SANYO, Osaka, Japan).

Cucumber plants at the second leaf stage were transferred to chilling stress treatment at 8 °C for 7 days, followed by recovery for 3 days under normal conditions. Control plants were grown at 28/25 °C day/night temperature. We used 10 plants per cultivar for stress treatment, and 10 plants were used as controls.

### 2.2. Electrolyte Leakage

Four leaf disks (d 10 mm) from the first fully developed leaf of each plant were immersed in 5 mL of deionized water. The electrical conductivity (E_1_) of the solution was measured by using a Mettler Toledo MC 22C conductivity meter (Greifensee, Switzerland) after 24 h of incubation at room temperature. The samples were then autoclaved, cooled, and the electrical conductivity (E_2_) was measured again. Results are calculated according to Dong et al. [31] as follows:

(1) $$\%\;\mathrm{Electrolyte}\;\mathrm{leakage}\;=\;\frac{E_{1}}{E_{2}}\;\times \;100$$

Samples were collected on days 3, 5, and 7 during low-temperature stress and in control growth conditions from 5 plants per treatment.

### 2.3. Measurement of Photosynthesis Rate

Leaf gas exchange parameters were measured on the first fully developed leaf using a CI-340 Handheld Photosynthesis System (CID BIO Science, Camas, WA, USA) infrared gas analyzer between 10 AM and 1 PM. Net photosynthesis rate (Pn) was determined by measuring the rate at which a known leaf area assimilates CO_2_ over time. The data for transpiration rate (E) and stomatal conductance (C) were also recorded. Measurements were taken on 0, 3, 5, and 7 days for both stressed and control plants, with 5 plants per treatment and 5 technical repetitions.

### 2.4. GC-MS Analysis of Primary and Secondary Metabolites

The first fully developed leaves from stressed and control plants were freeze-dried and used for Gas Chromatography coupled with Mass Spectrometry (GC/MS) analysis. Sample preparation, extraction, and derivatization of compounds were performed according to Zaharieva et al. [32]. GC-MS analysis was conducted on an Agilent GC 7890 gas chromatograph coupled with an Agilent MD 5975C mass spectrometer (Santa Clara, CA, USA). Separation of the compounds was carried out on an Agilent HP-5 ms column ((5%-phenyl)-methylpolysiloxane), using the following temperature program: initial temperature of 80 °C with a 2-min hold, ramped up to 200 °C at 5 °C per minute with another 2-min hold, and then increased to 300 °C at 10 °C per minute, maintaining that temperature for 10 min. A volume of 1 µL of each sample was injected in split mode (10:1) with the injector set to 250 °C. The mass spectrometer was operated at 200 °C, and the transfer line was kept at 250 °C. Electron impact ionization at 70 eV was used. To determine retention indices, a standard mixture of C10–C40 n-alkanes (Sigma-Aldrich, St. Louis, MO, USA) was analyzed using AMDIS version 2.73 (NIST, Gaithersburg, MD, USA). Compound identification was based on comparisons of Kovats retention indices and mass spectra with data from the NIST 08 library, the Golm Metabolome Database, and existing literature.

Measurements were taken on days 3, 5, and 7 for both stressed and control plants, with five independent biological replicates prepared from five plants per treatment.

### 2.5. Statistical Analysis

Student’s *t*-test was performed using IBM SPSS Statistics v. 27 (IBM, Armonk, NY, USA) for GC-MS data analysis of metabolites, measurement of photosynthesis activity, and electrolyte leakage.

### 2.6. Spectral Measurement

A portable, handheld MicroNIR OnSite-W instrument (Viavi Solutions, Santa Rosa, CA, USA) was used for spectral acquisition. The instrument setup was 100 scans per single spectrum and an integration time of 0.1 ms. After calibration of the instrument, spectra were measured from each plant on the cotyledons, 1st and 2nd true leaves. The instrument was positioned directly on the top leaf surface, and at least two measurements were taken at two different points on the leaf. A thick black plate is placed under the leaves during the measurement. Measurements were made from the first to the 7th day of low-temperature stress and on the 3rd day after recovery to 28 °C. A total of 140–150 spectra of each leaf of control and stressed plants were obtained, and 25–30 spectra of each leaf of recovery plants.

### 2.7. Spectral Data Processing

Pirouette v.4.5 software (Infometrix, Inc., Bothell, WA 98011, USA) was used for spectral data processing. Partial Least Squares (PLS) regression equations were developed for the quantitative determination of days of stress. The measured plants’ spectra were divided into a calibration data set (spectra of plant numbers 1, 2, 4, 5, 7, 8, and 10) and a validation set (spectra of plant numbers 3, 6, and 9). PLS models were developed with spectral data transformed as the second derivative and leave-one-out cross-validation. One sample from the calibration data set was excluded, and a model was developed using the remaining samples. This model was used to predict the tested parameter for the omitted sample. This procedure was repeated until each sample was used once as a validation sample, and the correlation coefficient between the predicted and reference values, as well as the standard error of cross-validation (SECV), was calculated. The prediction capacity of each calibration model was evaluated using statistical parameters from the calibration procedure: R, multiple correlation coefficient between reference values and NIR predicted values; standard error of calibration (SEC); and standard error of cross-validation (SECV). The number of latent variables in the models was selected based on the SECV values, which corresponds to the number at which the minimum SECV is obtained. The developed PLS models were validated with an independent validation set.

### 2.8. Aquaphotomics Analysis

An aquagram is a radar chart with coordinates connected with specific water absorption bands of free water, dimers, trimers, solvation shells, etc., named water matrix coordinates (WAMACs) [18]. To calculate the aquagram coefficients, the spectral data were first transformed using multiplicative scatter correction (MSC) to reduce scattering effects due to the different thicknesses and surface properties of the leaves. After that, normalized absorbance values at several wavelengths, *Aq*, were calculated using the equation:

(2) $$Aq_{\lambda }=\frac{A_{\lambda }-\mu_{\lambda }}{\sigma_{\lambda }}$$

where *A*_λ_ is the absorbance at a wavelength *λ* after multiplicative scatter correction (MSC) transformation of spectral data, *μ*_λ_ is the mean value of all spectra, and *σ*_λ_ is the standard deviation of all spectra at a wavelength *λ*, respectively. Aquagrams were calculated using the initial 12 WAMACs [23] and the new 7 WAMACs proposed by Vitalis et al. [29].

**Figure 1:**
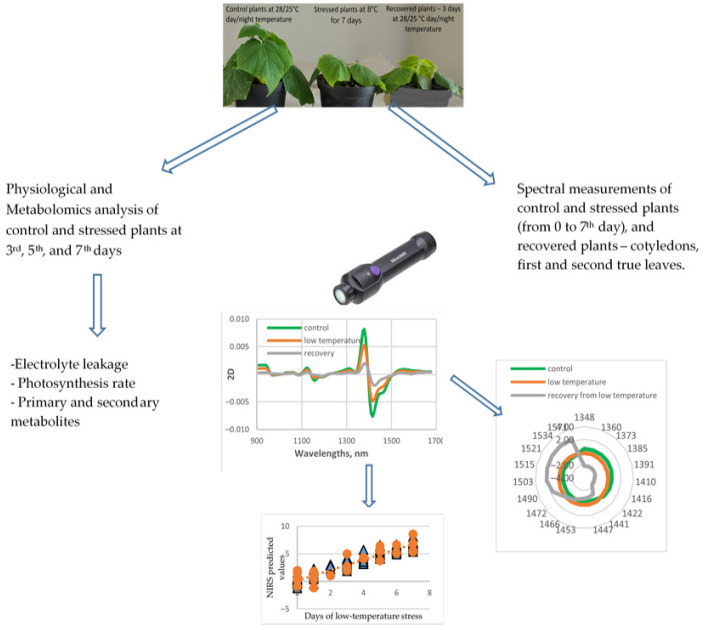
The scheme of experimental design.

The experimental design scheme is presented in Figure 1.

## 3. Results and Discussion

### 3.1. Effect of Chilling Stress on Electrolyte Leakage and Photosynthetic Activity

**Figure 2:**
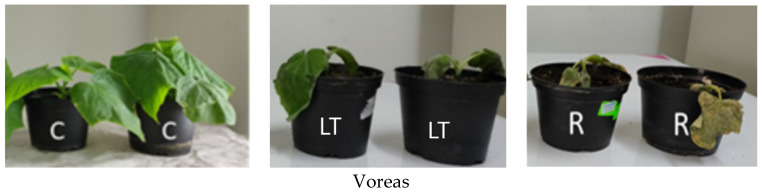

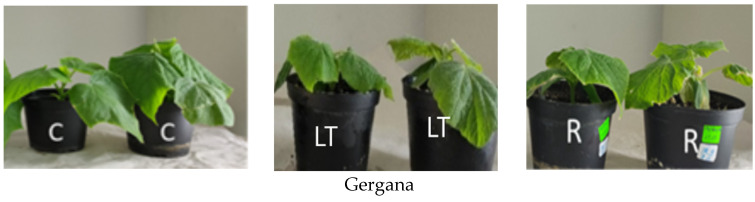
Phenotypic changes during low-temperature stress in cucumber cultivars Gergana and Voreas F1. C—control plants grown at 28 °C; LT—plants grown for 7 days at low temperature (8 °C); R—plants recovered for 3 days at 28 °C after low-temperature stress.

Cucumber cultivars Gergana and Voreas were subjected to chilling stress for 7 days and then returned to grow at normal temperatures. No damage was observed in the first days of low-temperature treatment in both varieties. Gergana plants showed slight symptoms of chilling injuries, wilting of leaves on day 7 of stress. Upon transfer to normal growth temperatures, the plants recovered. Conversely, low temperatures for 7 days caused wilting, water-soaked areas, and necrotic spots in the leaves of Voreas. None of the Voreas plants recovered after 7 days of chilling (Figure 2).

**Figure 3:**
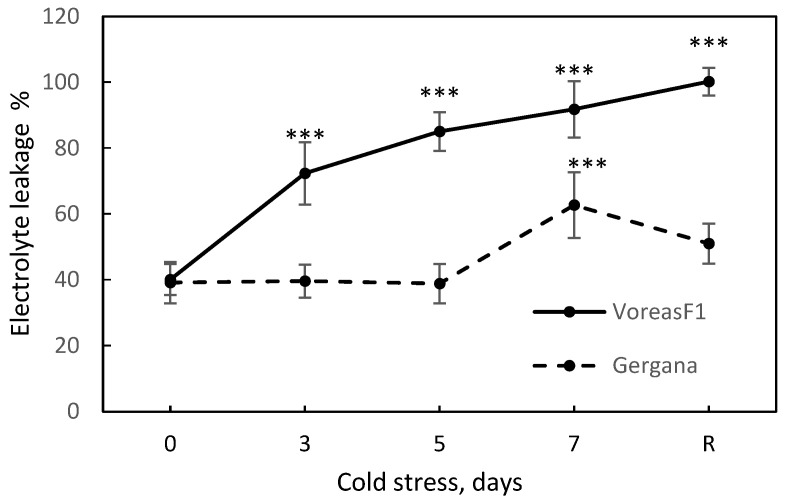
Electrolyte leakage during chilling stress and recovery of cucumber cv. Gergana and Voreas. Error bars represent the standard deviation. A *t*-test was applied to determine statistical differences between controls and the stress variants for each cultivar: *** *p* < 0.001.

Electrolyte leakage (EL) has been highlighted as a key physiological marker for assessment of low-temperature (LT)-induced damage in cucurbit plants [33]. We measured EL to evaluate cell membrane integrity during stress (Figure 3). In cv. Gergana, membranes remained stable until the 5th day of stress, showing a slight increase in EL at day 7, and then recovered at normal temperature. In cv. Voreas, EL from leaves increased at the third day at low temperature, reached 92% at the end of the treatment, and 100% at the recovery point, indicating complete disruption of the cell membranes.

**Figure 4:**
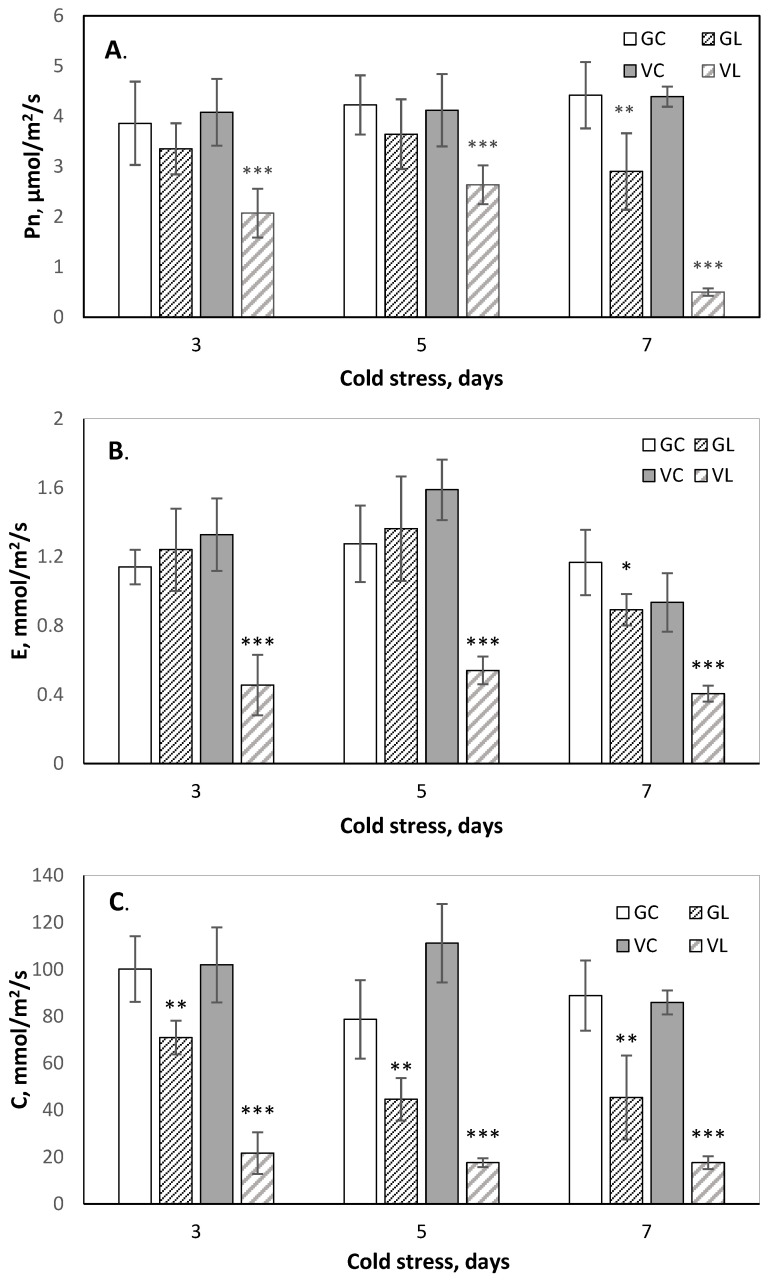
Photosynthesis efficiency during low-temperature stress of the cucumber cv. Gergana and Voreas. (**A**) Net photosynthesis rate—Pn; (**B**) Transpiration rate—E; and (**C**) Stomatal conductance—C; GC—Gergana controls; GL—Gergana at low-temperature stress; VC—Voreas controls; VL— Voreas at low-temperature stress. Error bars represent the standard deviation. A *t*-test was applied to determine statistical differences between controls and the stress variants for each cultivar: * *p* < 0.05; ** *p* < 0.01; *** *p* < 0.001.

An infrared gas analyzer was used to evaluate chilling stress-induced damage to photosynthetic efficiency in cucumber plants as an indicator commonly used in plant physiology for assessment of the impact of abiotic stress on plant growth [6,12]. At normal growth temperatures, both cultivars showed similar values for all studied photosynthesis parameters, and no statistically significant differences were detected between cultivars. However, at LT treatment, the photosystems of each cultivar showed distinct behavior. The cv. Gergana maintained a nearly constant net photosynthesis rate (Pn) during LT treatment with only a slight decrease (30%) compared to controls at the 7th day (Figure 4A). In Voreas, Pn decreased from about 50% on the third and fifth days at LT to 90% on the 7th day. Other gas exchange parameters measured—transpiration rate (E) and stomatal conductance (C)—showed a correlation with the dynamic changes in Pn during stress (Figure 4B,C). Low photosynthetic efficiency in cv. Voreas during chilling stress is related to stomata closure and lack of transpiration. There is a small but statistically significant reduction in stomatal conductance and steady transpiration rate during LT treatment in cv. Gergana. These results align well with the phenotypic differences observed between both cultivars and reveal better adaptability of Gergana.

### 3.2. Metabolomics Changes During Chilling Stress

GC/MS analyses revealed changes in plant metabolites at LT stress (Table 1). During chilling conditions, the content of most metabolites was reduced compared to controls, and this was observed from day three until the end of the LT stress. Soluble sugars are key components in plant adaptation to chilling temperatures, playing the role of osmoprotectants on one hand and maintaining cell membrane stability on the other [34]. In both varieties, we observed a decrease in sugars under LT treatment, more pronounced in Voreas, where the sucrose is almost completely depleted at the 7th day of cold. The organic acid content decreased slightly in both cultivars. The fatty acid content at the 7th day of LT treatment was the same as in controls or slightly increased. The main difference in the metabolic profile of the two cultivars is related to phenolic acids. In Voreas, phenolic acids decreased from the third day and remained lower compared to untreated plants. In Gergana, the phenolic acid content did not change significantly (Ferulic acid) or accumulate by a 2-fold increase in cinnamic acid by the seventh day. Phenolic compounds possess antioxidant properties and play a crucial role in scavenging reactive oxygen species and maintaining membrane stability during abiotic stress [35,36,37]. Accumulation of these protective compounds as a response to LT stress correlates with alleviated chilling injuries observed in cv. Gergana compared to Voreas plants.

### 3.3. Near-Infrared Spectra of the Cucumber Plant During Cold Stress

**Figure 5:**
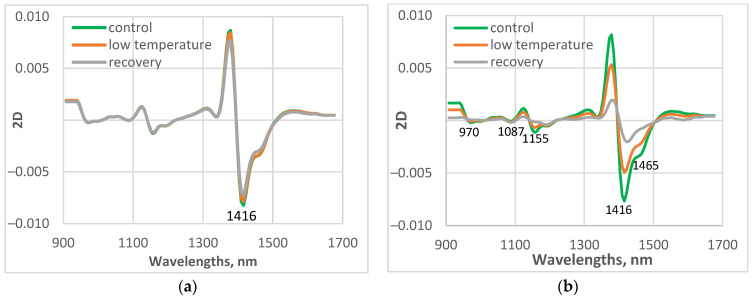
Average values of spectral characteristics of the studied control, low-temperature treated, and recovered plants, transformed as the second derivative (2D). (**a**) cv. Gergana, first leaf; (**b**) cv. Voreas, first leaf.

Average spectral characteristics of the first leaf of the studied control, low-temperature-grown, and recovered plants transformed as a second derivative (2D) are presented in Figure 5. The second derivative transformation allowed the separation of overlapping peaks that are difficult to distinguish in the original spectrum, making small or hidden peaks more visible, especially useful for detecting minor compounds.

The largest maximum was observed at 1416 nm, due to the absorption of free water. The spectral features in the region 1450–1470 nm depend mainly on different water molecular conformations. Hydrogen bonding strongly shapes the NIR spectrum of water by broadening absorption bands and shifting their positions. The strength of the hydrogen bonding influenced the position of the O–H stretching vibration. Stronger hydrogen bonding shifted the maximum to a higher wavelength. Wang et al. [38] used the wavelet transformation to investigate the structure of water in aqueous systems. The authors reported in the transformed overlapped broad peak of water around 6900 cm^−1^ (1449 nm) three peaks at 7111, 6966, and 6841 cm^−1^ (1406, 1436, and 1462 nm), which can be assigned as vibration of non–hydrogen–bonded, weakly hydrogen-bonded, and strongly hydrogen–bonded OH in a water molecule, respectively.

Structured layers of water molecules surrounded proteins in plants. These hydration shells also alter the O-H overtone bands [39,40]. Tsenkova et al. [41] discussed water–pion interaction at 1464 nm and showed a difference in water conformation around the same protein with different metals in the octapeptide reaction. Ma et al. [42] investigated the function of water during the gelation of globular proteins. The results indicated that in native and molten globule states, water species with two hydrogen bonds (around 1466 nm) are located in the hydration shell of proteins to maintain the stability of their structure.

Another process that could influence water molecular structure is ion hydration. Electrolyte leakage in plants leads to the loss of ions. In cold stress, the most essential ions involved in electrolyte leakage were potassium and calcium ions from the plant tissue [37]. Ion hydration influences spectra because hydrated ions alter the hydrogen-bonding network of water, shifting absorption bands and intensities. Uchida et al. [43] investigated the interaction of water and dissolved salts. The results suggested that water spectral variations could be explained more likely with changes in the hydrogen-bonded network than by the direct interaction between ions and water molecules. Tsenkova et al. [44] presented a review of the investigations of the effects of low concentrations of salts on water structure. Each salt and concentration level affected the water differently.

Another small absorption band was at 970 nm, 1087 nm, and 1155 nm. The bands at these wavelengths were connected with the absorption of the O-H group [45].

The spectral patterns of control plants from the two studied cucumber varieties were very similar. The differences in the spectral characteristics of the stressed and recovered plants were clearly visible between the two cucumber varieties. The differences between the spectra of control and treated plants were much smaller for cv. Gergana compared to those for Voreas. The results are similar for the other measured leaves.

**Figure 6:**
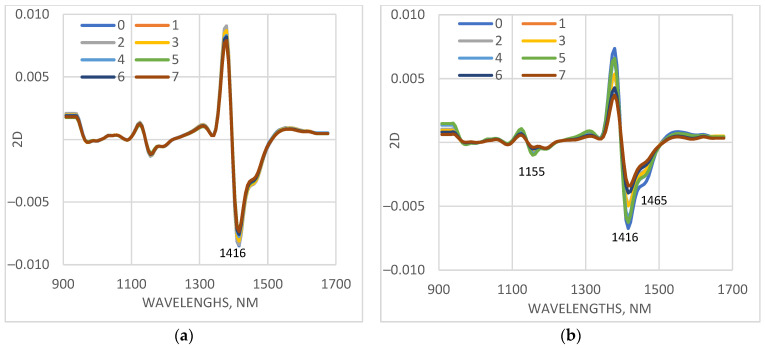
Average values of the spectral characteristics of first leaves of plants grown under low-temperature stress, transformed as a second derivative (2D), measured on different days (from 0 to 7th days of measurement). (**a**) cv. Gergana, first leaf; (**b**) cv. Voreas, first leaf.

Spectra of only stressed plants are presented in Figure 6. The most significant differences in plants of the cv. Voreas, depending on the days of LT treatment, are observed in the range of 1410–1478 nm. The absorption of leaves of the cv. Voreas in this range decreases significantly with the increasing days of exposure to low temperature. Another wavelength range with an observed difference in absorption was around 1155 nm. The differences among the spectra of cv. Gergana measured on different days of stress treatment were much smaller compared to those for Voreas. Similar results were obtained for the other measured leaves.

The spectral differences confirmed the results of electrolyte leakage and photosynthetic activity determination (Figure 3 and Figure 4). Gergana cultivar plants retained cell membrane integrity and photosynthesis rate, with slight shifts compared to the controls at day 7 of LT treatment. Small physiological changes result in minor alterations in the spectral characteristics of the measured leaves.

Electrolyte leakage from leaves of the Voreas cultivar increased throughout the period of low temperature and reached 92% on the seventh day. The photosynthesis rate for the Voreas cultivar plants decreased to 90% on the 7th day. This explains the significant differences in the spectra measured on different days of low-temperature exposure.

**Figure 7:**
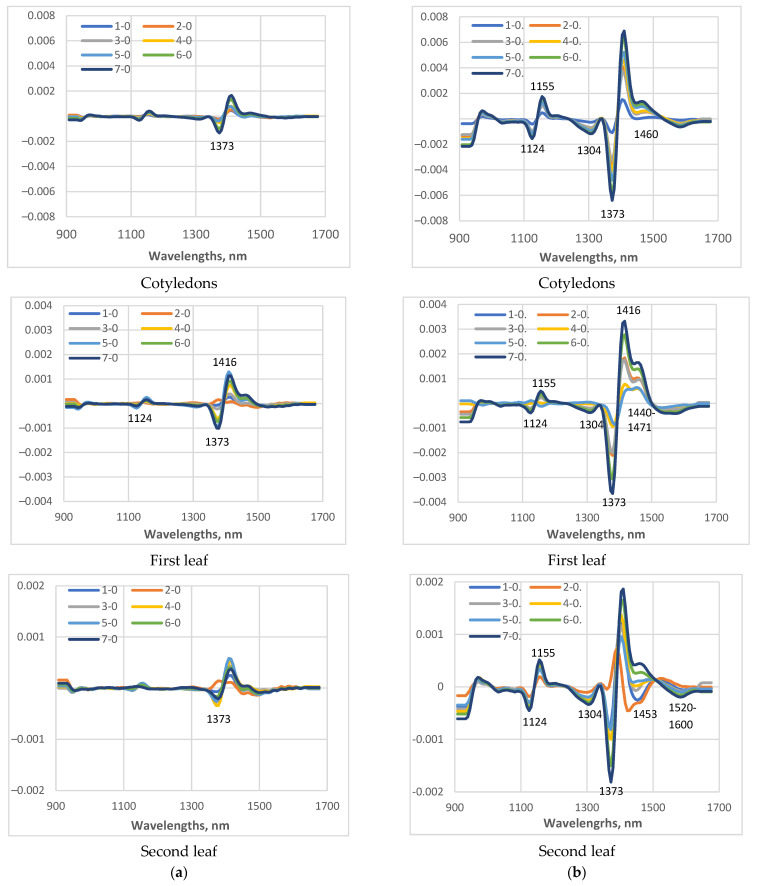
Differences between the spectral characteristics transformed as the second derivative of plants grown under low-temperature stress, measured on different days (days from 1st to 7th), and the spectra of the control (zero day). In figures, the legend 1-0 means spectra measured at day 1 minus spectra at day 0; 2-0 means spectra measured at day 2 minus spectra at day 0, etc. (**a**) Gergana cultivar; (**b**) Voreas cultivar.

For a more detailed study of the changes in the spectral characteristics of the studied cucumber plants under low-temperature stress, the differences in the average spectra measured on day 0 and on days 1 to 7 of the LT stress were calculated (Figure 7). With the same scale of the coordinate axes for a particular leaf, it is clearly visible how much smaller the differences were in the plants of cv. Gergana compared to those for cv. Voreas, indicating greater low-temperature-induced changes in the plants of cv Voreas. When comparing the results for cotyledons, first leaf, and second leaf, differences were again observed. In plants of cv. Gergana, the differences between the spectra of control and stressed plants for cotyledons and the first leaf were approximately the same, and for the second leaf, they were smaller.

For plants of cv. Voreas, the most significant differences were observed for the cotyledons, followed by the first leaf, and the smallest for the second leaf. The larger observed differences in cotyledons can be explained by their different structure and different water content compared to those of the first and second true leaves.

The main differences between the spectra of stressed plants are observed around 1373 nm. This wavelength is one of the initially selected WAMAC, marked as c3 [23]. In the investigation related to changes during the storage of ready-to-eat rocket [46], the significance of absorbance at 1373 nm for separation between fresh and longer cold-stored samples was reported.

The other regions where differences were observed were in the ranges around 1124 nm, 1304 nm, 1440–1500 nm, and 1520–1600 nm. The absorption in 1440–1500 nm is dominated by water absorption. The range 1520–1600 nm indicated a higher content of bound water. Investigation of water interaction with polymer matrices was reported by Moll et al. [47]. More hydrophilic polymers, such as biopolymers, showed peaks in the vicinity of water bands, indicating the presence of water bound to polymers. Schwanninger et al. [48] also reported bands located at that region to H-bonded O–H groups of cellulose. Again, the differences in 1440–1500 nm and 1520–1600 nm were much greater for the Voreas cultivar compared to those of the Gergana cultivar.

### 3.4. Aquagram Analysis

**Figure 8:**
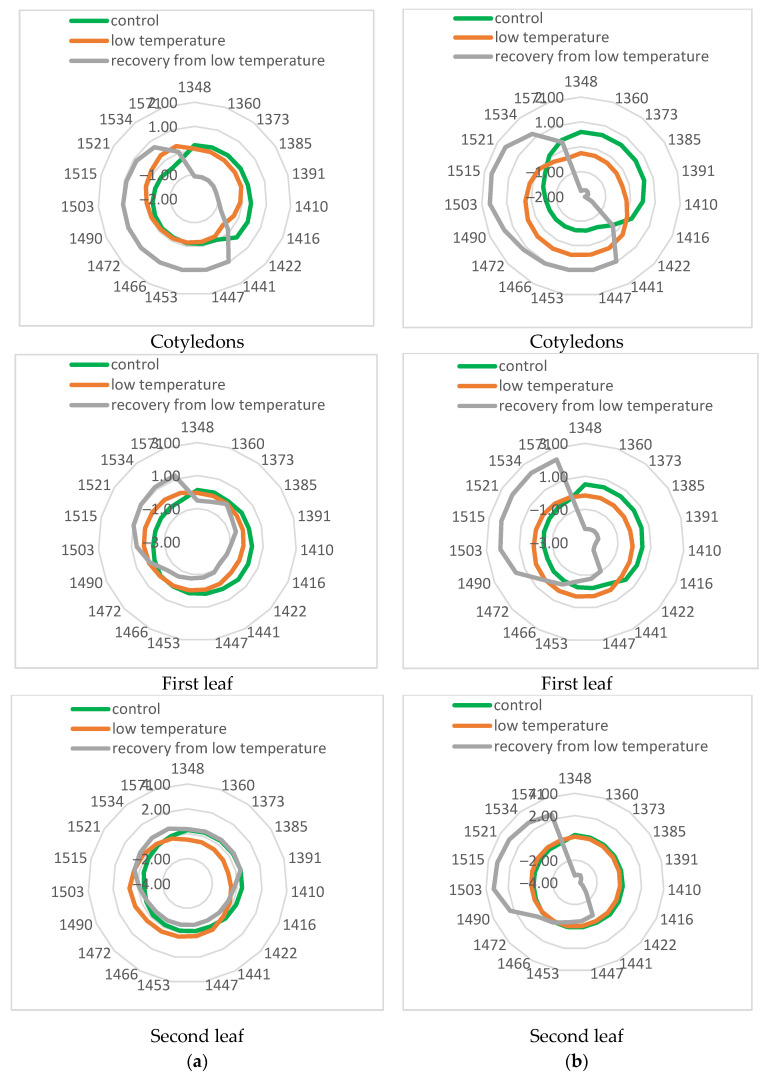
Aquagrams of control, stressed, and recovered cucumber plants. (**a**) cv. Gergana; (**b**) cv. Voreas.

To further investigate the changes in leaves during low-temperature stress, aquagrams were calculated for control, stressed, and recovered plants (Figure 8). The aquagrams showed that the two investigated cucumber cultivars responded differently to the cold stress.

The aquagram for the cotyledons of the cv. Voreas showed apparent differences between control, stressed, and recovered plants. Aquagram values in the range 1348–1416 nm of control plants were higher than those of stressed plants, while in the range 1422–1503 nm, those for stressed plants were higher. This means that in stressed plants, the water molecular structure changes. According to WAMAC’s assignments, this means that in stressed plants, strongly bound water (molecules with 2 hydrogen bonds at 1466 nm, with 3 hydrogen bonds at 1478 nm, and with 4 hydrogen bonds at 1490 nm) increased. The same trend, but to a lesser extent, is observed for the first leaf. The differences in aquagrams between the second leaf of the control and stressed plants are much smaller. Very different was the aquagram of the Voreas cultivar for recovery from low-temperature plants. There was a sharp decrease in the coefficients of aquagram in the 1348–1422 nm region and an increase in the 1453–1571 nm region. Slight differences in the aquagram of recovery of Voreas plants were observed between cotyledons and first and second leaves at WAMAC 1534 and 1571 nm, which could be connected with water-cellulose and water-protein interactions. The changes in aquagrams indirectly confirmed the results of electrolyte leakage and photosynthesis efficiency for Voreas as well as spectral changes during low-temperature stress.

The aquagrams of cv. Gergana showed some different characteristics compared to those of Voreas. More similar to those of Voreas were aquagrams for cotyledons, especially aquagrams for recovery plants. Aquagrams of recovery plants for the first and second leaves were different from those of Voreas. There is no such decrease in the aquagram coefficients in Gergana’s first leaf at WAMAC’s 1373 and 1385 nm, as is visible in Voreas plants. Only small differences were found among aquagrams for the second leaves. The aquagrams also showed that the changes caused by LT treatment in cotyledons of both studied cultivars were bigger than for the second leaf, probably caused by differences in water content between them.

Aquagrams confirmed differences between the two investigated cucumber cultivars related to tolerance to low temperatures. Aquaphotomics could be used as an additional approach for the investigation of the low-temperature tolerance of cucumber cultivars.

**Figure 9:**
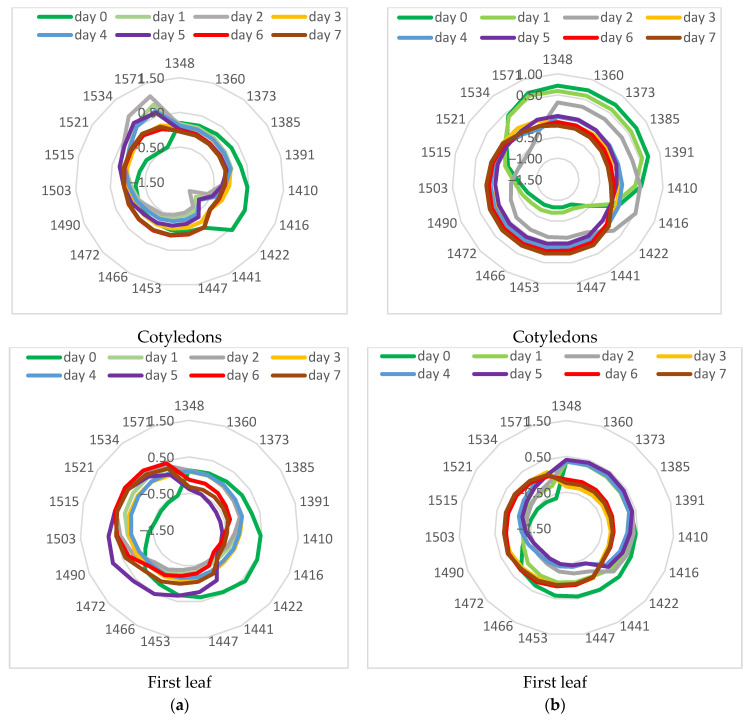
Aquagrams of stressed cucumber plants. (**a**) cv. Gergana; (**b**) cv. Voreas.

Aquagrams based on the spectral characteristics of the studied treated leaves, measured on different days of LT treatment, were calculated to study the dynamics of changes in their water state. Aquagrams for the cotyledons and for the first leaf of plants of the cv. Gergana and Voreas are presented in Figure 9.

There is a significant difference between the aquagram of initial day 0 and days 1 and 2 for Gergana cotyledons. The values in the region of 1410–1441 nm decreased, while the aquagram coefficients in the 1521–1571 nm range increased, indicating a change in the water molecular structure. In the aquaphotomics investigation, the region 1521–1571 nm was interpreted as strongly bound water related to water–polymer interaction [30]. Vitalis et al. [29], in the investigation of lettuce cold storage, identified 1503, 1521, 1534, and 1571 nm as WAMACs and concluded that increased absorbance in this region reflected loss of free and weakly bound water and a predominance of water bonded to the structural elements such as cellulose and other polymers in the tissue. Moyankova et al. [28] also reported increased coefficients in aquagrams in those regions in drought-stressed maize plants. Together, these aquaphotomics studies provided consistent evidence that in plant tissues the 1503–1571 nm range reflected strongly bound, polymer-associated water, particularly water associated with cellulose and other structural polysaccharides, rather than free water.

A characteristic decrease is observed at 1442 nm for Gergana leaves. This is not observed in aquagrams for Voreas plants. Kuroki et al. [49] reported differences in absorption at an area of about 1440 nm between the resurrection plant with an extremely high desiccation tolerance and non-resurrection plant species. The absorption at 1442 nm is strongly influenced by hydrogen bonding. The authors assigned absorption at 1440 nm to water molecules with one hydrogen bond (dimers). This confirmed differences in the reaction of the investigated cucumber plants to LT stress.

For the first leaf, again, the most significant differences are observed between day zero and the first and second days. We observe a sharp decrease in the values obtained for the first and second days of the LT treatment in the range of 1410–1447 nm and an increase in the range of 1503–1571 nm.

The fastest changes in the cotyledons of the Voreas cultivar are observed between the control and the second day of low-temperature stress. The aquagrams between the fourth and seventh days are similar in shape. A clear trend is seen for decreasing aquagram values in the 1348–1410 nm range and increasing them in the 1447–1503 nm range.

Slightly different were the dynamics of changes in aquagrams for the first leaf of Voreas plants. The biggest differences were observed between the control and the first two days of low-temperature stress, but in different WAMACS between 1422 and 1472 nm. A grouping of the aquagrams is observed for the fourth and fifth, as well as for the sixth and seventh days. Surprisingly, the aquagram for the third day of low-temperature stress is closer to those for the sixth and seventh days than for the fourth and fifth. Probably, there are some unstable changes in the water structure on the third day.

### 3.5. PLS Models for the Determination of Days of Cold Stress

PLS models were created to determine the days of low-temperature stress using the spectral range from 900 to 1700 nm, and their statistical parameters for calibration and validation data sets are presented in Table 2 for Gergana and Table 3 for Voreas. The cross-validation errors of determination of the days of low-temperature stress were from 0.67 to 1.04 days for plants of the Gergana cultivar and from 0.98 to 1.14 days for the Voreas cultivar, depending on the spectral information on different leaves used. The accuracy of determination for the independent set was similar to that of cross-validation.

**Figure 10:**
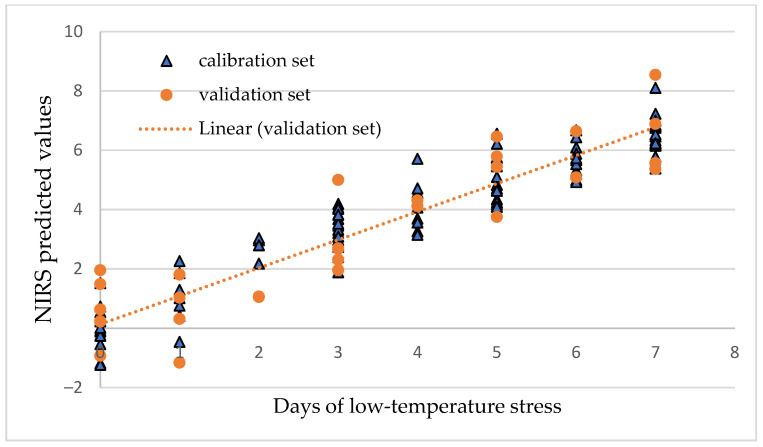
PLS regression model for the determination of days of cold stress of Gergana plants, first leaf.

A graphical illustration of the accuracy of the determination of days of cold stress for the first leaf of Gergana plants is presented in Figure 10.

**Figure 11:**
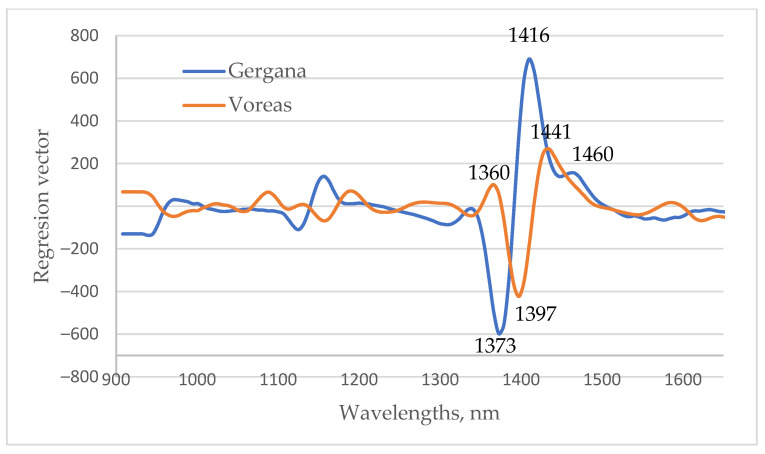
Regression vector from PLS regression equations for the determination of days of low-temperature stress for the first leaf.

An illustration of the regression vectors from PLS regression models used to determine the days of low-temperature stress for the first leaf is presented in Figure 11. The most significant wavelengths were in the 1350–1470 nm regions, respectively. A similar finding was observed for models based on the spectra of cucumbers’ cotyledons and second leaves. The wavelengths with the biggest coefficient in the regression vector for the determination of days of low-temperature stress for cv. Gergana corresponds precisely to the wavelengths at which the greatest differences are observed in the spectra of plants under LT stress. Similarly to Gergana plants, the range of wavelengths in which the coefficients in the regression vector for Voreas were the biggest was in the area where differences in the spectral characteristics of plants measured on different days of stress were observed. The regression vectors confirmed the importance of the region of water absorption for low-temperature stress detection in cucumber plants. The significant wavelengths at 1360, 1373, 1416, and 1441 nm correspond exactly to some of the WAMAC.

## 4. Conclusions

This study was conducted to investigate the changes in two cucumber cultivars—Gergana and Voreas F1—under low-temperature stress using near-infrared spectroscopy and an aquaphotomic approach and to compare the outcomes derived by both physiologic and spectral analyses. Results obtained via conventional biochemical methods showed that cv. Gergana was cold-tolerant and maintained cell membrane integrity and photosynthesis activity during low-temperature stress. Voreas F1 plants were unable to survive seven days of low-temperature stress due to a drastic increase in electrolyte leakage and reduction in the net photosynthesis rate, stomatal conductance, and transpiration rate.

Differences in near-infrared absorption spectra of cucumber leaves were observed between control, stressed, and recovered plants, as well as between different days of stress. The most significant differences were in the range of the first overtone of water, from 1300 to 1600 nm. These differences could be connected to changes in physiological and metabolic data. Aquagrams of the two studied cultivars also reveal the differences in their response to low temperatures and changes in the water molecular structure in leaves. It is possible to create PLS models for determining the days of low-temperature stress based on the spectral characteristics of leaves. These results can be used as a fast and non-destructive approach for the investigation of low-temperature stress in cucumber cultivars.

## Figures and Tables

**Table 1:** Heat map of metabolite changes in cucumber plants cv. Gergana (A) and cv. Voreas (B) during chilling stress—3rd, 5th, and 7th days of low-temperature treatments and control plants. The relative metabolite concentrations are expressed as log2, normalized to controls. (blue–white–red heat map). Blue and red colors indicate a low and high metabolite level, respectively.

	Gergana				Voreas F1			
Metabolites	Control	3 Days	5 Days	7 Days	Control	3 Days	5 Days	7 Days
**Sugars**								
D-Fructose sum	**0**	−1.7 *	−2.2 *	**−0.2**	**0**	−1.7 **	−1.1 *	−2.7 * *
D-Glucose	**0**	−2.5 ***	−2.6 ***	**−0.8**	**0**	−2.8 *	−2.9 *	−3.4 *
Sucrose	**0**	−2.1 *	−3.8 *	−2.2 *	**0**	−3.3 **	−0.5 *	−6.7 * *
Myo-Inositol	**0**	**−0.2**	**−0.1**	**0.1**	**0**	**0.2**	**0.3**	**0.1**
**Alcohols**								
Glycerol	**0**	**0.2**	**0.2**	−0.5 *	**0**	**0.5**	**0.6**	**−0.1**
Galactinol	**0**	−0.9 * *	−1.0 *	−2.5 * *	**0**	−1.5 * *	−1.6 * *	−2.2 * **
**Organic acids**								
Lactic acid	**0**	**0.0**	**0.0**	**−0.1**	**0**	**0.1**	**0.0**	**−0.2**
Glycolic acid	**0**	**−0.3**	**−0.2**	**−0.3**	**0**	−0.6 * *	−0.5 * *	−0.9 * **
Succinic acid	**0**	**−0.1**	−0.8 * **	−0.8 * **	**0**	−0.6 *	**−0.3**	−0.8 *
Glyceric acid	**0**	−2.1 * **	−1.9 * **	−2.4 * **	**0**	−0.7 *	**−0.6**	−1.4 * **
Fumaric acid	**0**	**−0.1**	**−0.5**	−0.9 * *	**0**	−1.6 *	**−1.2**	−2.0 *
Malic acid	**0**	−1.6 * *	−2.7 * *	−2.9 * *	**0**	−2.1 * *	−2.9 * *	−3.2 * **
**Phenolic acids**								
Cinnamic acid	**0**	**0.1**	−0.8 *	1.1 * *	**0**	−0.8 *	−1.9 * *	−1.5 *
Ferulic acid	**0**	**0.1**	**0.4**	**0.2**	**0**	−1.2 *	−1.6 *	**n.d.**
**Fatty acids**								
Palmitic acid	**0**	**−0.2**	**0.2**	**0.2**	**0**	**0.5**	0.8 * *	**0.1**
Stearic acid	**0**	**0.4**	**0.2**	**−0.2**	**0**	1.1 * *	1.4 * *	0.5 *
1-Monopalmitin	**0**	**−0.6**	**−0.5**	−3.7 *	**0**	**−0.2**	**−0.8**	**−1.7**
Glycerol Monostearate	**0**	0.4 *	**0.4**	**−1.1**	**0**	**−0.2**	**−0.4**	−4.0 *

A *t*-test was applied to determine statistical differences between controls and the stress variants for each metabolite: * *p* < 0.05; ** *p* < 0.01; *** *p* < 0.001.

**Table 2:** Statistical parameters of PLS models for determining days of low-temperature stress, Gergana cultivars.

Leaves	Calibration Set					Validation Set	
	PLS Factors	SECV	Rcv	SEC	Rcal	SEP	Rval
Cotyledons	5	0.67	0.96	0.63	0.97	1.04	0.91
First leaf	2	0.81	0.94	0.80	0.95	1.03	0.91
Second leaf	3	1.04	0.91	0.99	0.92	1.03	0.91

Rcval, Rcal, and Rval—multiple correlation coefficients between reference values and NIR predicted values for cross-validation, calibration, and validation, respectively; SEC—standard error of calibration; SECV—standard error of cross-validation; SEP—standard error of prediction.

**Table 3:** Statistical parameters of PLS models for determining days of low-temperature stress, Voreas cultivars.

Leaves	Calibration Set					Validation Set	
	PLS Factors	SECV	Rcv	SEC	Rcal	SEP	Rval
Cotyledons	2	1.14	0.84	1.07	0.87	1.14	0.86
First leaf	2	0.98	0.89	0.96	0.90	1.04	0.85
Second leaf	7	1.03	0.89	0.89	0.92	0.97	0.89

Rcval, Rcal, and Rval—multiple correlation coefficients between reference values and NIR predicted values for cross-validation, calibration, and validation, respectively; SEC—standard error of calibration; SECV—standard error of cross-validation; SEP—standard error of prediction.

## Data Availability

All data are available from the corresponding author upon request. Spectra of the samples are available from the authors.

## Abbreviations

The following abbreviations are used in this manuscript:

NIR	Near-infrared
LT	Low temperature
Pn	Net photosynthesis rate
E	Transpiration rate
EL	Electrolyte leakage
C	Stomatal conductance
PLS	Partial Least Squares regression
Rcal	Multiple correlation coefficients between reference values and NIR predicted values calibration
SEC	Standard error of calibration
Rcv	Multiple correlation coefficients between reference values and NIR predicted values for cross-validation
SECV	Standard error of cross-validation
SEP	Standard error of prediction
